# Adults’ and children’s reasoning about the potential of diverse groups

**DOI:** 10.3389/fpsyg.2024.1455392

**Published:** 2024-11-13

**Authors:** Drew Weatherhead, Shaylene E. Nancekivell, Rebeka Workye

**Affiliations:** ^1^Department of Psychology & Neuroscience, Dalhousie University, Halifax, NS, Canada; ^2^Department of Psychology, University of Manitoba, Winnipeg, MB, Canada

**Keywords:** social cognition, diversity, innovative potential, cooperative potential, children

## Abstract

**Introduction:**

In two experiments, we examine the degree to which adults (Experiment 1) and children 5-to-8-years-old (Experiment 2) use diversity to infer a group’s cooperative and innovative potential.

**Methods:**

Participants heard a child-friendly vignette about a competition in which a homogenous and diverse group were competing to design the perfect toy. They were then probed using questions related to the group’s innovative potential and cooperative potential and asked to justify their responses.

**Results:**

Results show that adults believed that the diverse group would produce the more innovative product, while children endorsed the homogenous group. When asked a question probing cooperation, adults selected the homogenous group, whereas children were equally likely to select either group. Analysis of adults’ explanations showed their explanations differed depending on which group they endorsed. However, children’s explanations did not show this nuance. Exploratory analyses suggest that participants’ responses were influenced by their personal experiences with gender and racial diversity.

**Discussion:**

People’s appreciation of the link between group diversity and group potential changes across the lifespan likely due to life experiences.

## Introduction

Extensive prior work illustrates that group diversity is linked with positive outcomes. More demographically diverse groups tend to engage in more innovative behavior (Bantel and Jackson, 1989; Hambrick et al., 1996; Drach-Zahavy and Somech, 2001). This is likely because of how group members with differing demographic characteristics index different knowledge, skills, values, and beliefs (McGrath et al., 1995). Thus, as demographic diversity increases, the group’s cognitive diversity does as well (Bantel and Jackson, 1989; Watson et al., 1993; Jackson and Ruderman, 1995) but see counter discussion (Hambrick and Mason, 1984). Some have argued that diverse groups possess more creative potential and therefore have a performance advantage over homogenous groups (McLeod et al., 1996). For example, gender diversity has been associated with more innovative and creative ideas (Ruiz-Jiménez et al., 2016). Building on this prior work, the present study aims to understand how group composition, in terms of gender and racial diversity, influences adults’ and young children’s *beliefs* about a group’s cooperative and innovative potential.

Why map people’s beliefs about diverse groups? In other contexts, adults’ beliefs about group diversity have consequences for group functioning. In general, if group members hold pro-diversity beliefs, even a diverse group with a clear “us-them” subgroup distinction may perform better than a homogenous group (Homan et al., 2007). Related work suggests the innovative potential of diverse teams is dependent on people’s willingness to cooperate with others in the group (Roh and Koo, 2019) which is influenced by beliefs like whether they feel they are valued members of their team (Leroy et al., 2022). Beliefs about diversity in the workplace, including one’s openness to diversity, influences work relationships and the quantity of conflict in a work group (Hobman et al., 2003).

In more recent history, there is explicit messaging given to adults touting the benefits of diversity for creativity and innovation. For example, the increasing Justice Equity Diversity Inclusion and Accessibility (JEDIA) initiatives both in academia and industry stress the need for team diversity (e.g., Kohl, 2021). The present study addresses whether, and when, in development, intuitive theories about diversity might come to match these societal messages. To investigate this question, we compare the beliefs of young school aged children to adults. One possibility is that current societal messages reflect early emerging beliefs about the value of diversity. Namely, children from a young age understand that enhanced diversity leads to enhanced diversity of ideas. In this case, young children might focus on the links between experience and ideas, and less about the social composition of the group, when thinking about innovation. Alternatively, children might favor a homogenous ingroup due to a strong early emerging group psychology that heavily weights the similarities between group members. In this case, extensive societal messaging around diversity may be reflecting a need to educate people as they age about the benefits of diversity.

Across childhood, there is a relation between children’s beliefs about groups and their behavior, broadly speaking (e.g., Rhodes, 2012; Rhodes et al., 2017). While beliefs about “diverse groups” have not been directly studied, there has been extensive work focused on children’s understanding of how group membership influences preferences (i.e., ingroup preferences), as well as children’s normative beliefs about how highly similar or homogenous group members should behave towards one another.

First, from a young age, people prefer ingroup members over outgroup members when considering multiple kinds of homogenous groups (Aboud, 1988; Kinzler et al., 2007; Dunham, 2018; deMayo and Olson, 2024). For example, when asked to choose a new friend or rate individuals on a Likert scale, children generally prefer members of their own social groups relative to members of outgroups (Dunham, 2018; deMayo and Olson, 2024). By 5-years-old, social preferences for people of the same sex/gender are very strong, with children preferring to interact with same-gendered peers (Maccoby and Jacklin, 1987; deMayo and Olson, 2024), and prefer stereotypically gendered clothing consistent with their self-identified gender (Halim et al., 2014; Fast and Olson, 2018). In contrast, children’s race-based social preferences are typically less pronounced than that of accent and language or sex/gender (deMayo and Olson, 2024). For example, when two social properties are pit against each other (i.e., the choice of an individual who speaks your native language but is of a different race or speaks an unfamiliar language but is the same race) children prioritize language (Kinzler et al., 2009). Nonetheless, when race is presented alone, children will choose same-race friends (Aboud, 1988; Dunham et al., 2006; Kinzler and Spelke, 2011). Importantly, children’s essentialist beliefs (i.e., that certain social properties like gender and race are stable and unchanging) are linked to their preferences for ingroups and biases toward outgroups. Indeed, stronger essentialism is positively correlated with out-group stereotyping in young children (Levy and Dweck, 1999; Pauker et al., 2010; Gaither et al., 2014). For example, 3-to-10-year-olds’ essentialist beliefs about race predicted children’s endorsement of negative racial stereotypes (Pauker et al., 2010) consistent with adult behavior (Hoffman and Hurst, 1990; Martin and Parker, 1995; Bastian and Haslam, 2006), and when essentialist beliefs are induced in 4-to-6-year-olds, they share fewer resources with members of essentialized out-groups (Rhodes et al., 2017).

A second related literature has looked at children’s expectations of the behavior of group members who belong to homogenous groups. This work suggests that children view social categories as having both descriptive characteristics, that is, group members typically share common characteristics or traits (e.g., noticing group members speak a certain way or are from a similar place;^;^ Weatherhead et al., 2016, 2018), and endorse prescriptive norms, that is, they think that these characteristics ought or should be shared by individuals belonging to the same group (e.g., thinking group members *should* wear a specific style of clothing; Trautner et al., 2005; Roberts et al., 2017a, 2017b, 2018; Roberts and Horii, 2019). Critically, these two types of beliefs are often conflated by adults and children, with descriptive beliefs often leading to prescriptive beliefs. Other expectations children have for group members’ interactions include that they will share both physical and knowledge-based resources, and that they will follow similar social-cultural conventions (Wen et al., 2016; Yu et al., 2016; Gonzalez et al., 2020; Liberman et al., 2020).

In sum, children and adults prefer ingroup members over outgroup members and have strong expectations for how ingroup members should behave. To our knowledge, no existing literature has examined how group diversity influences people’s thinking about the group’s potential, and whether it aligns with well documented positive behavioral outcomes related to a group’s potential for innovation and cooperation. The current study fills this gap in the literature by evaluating whether children and adults are sensitive to group diversity when making inferences about group’s potential.

The current study maps people’s thinking about the benefits of group diversity including how it might change from childhood to adulthood. Specifically, we ask to what degree both adults’ (Experiment 1) and young children, aged 5- to 8-years-old, (Experiment 2) use group diversity and homogeneity to understand a group’s cooperative and innovative potential. 5- to −8-year-olds were of particular interest as by 5-years-old children demonstrate strong social preferences for individuals of the same race and same gender. Potential in this context refers to the group’s capacity to exhibit a certain property. Innovative potential refers to the group’s capacity to creative an innovative product, while cooperative potential refers to the group’s capacity to work together in a harmonious manner. The scope of the term “group” in this study is restricted to that of a somewhat arbitrary team. This is meant to reflect the workplace setting in which previous work has investigated the benefits of diversity (e.g., Bantel and Jackson, 1989; Hambrick et al., 1996; Drach-Zahavy and Somech, 2001). We explicitly gave the groups labels and matching t-shirts in our stimuli because previous work has demonstrated children infer categorical boundaries when group membership is denoted by labels (i.e., minimal group membership; Dunham et al., 2011; Gelman and Heyman, 1999; Master and Walton, 2013; Waxman, 2013). We focus on how beliefs about group potential might change with age because of the importance of understanding developmental trajectories in determining the role of external influences on people’s perspectives across the lifespan.

Because of the interest of mapping development, all participants heard a child-friendly vignette about a toy competition in which a homogenous and diverse group were competing to design the perfect toy. The diverse group members varied in race, gender, and physical traits (e.g., height), whereas the homogenous group did not vary in any of these dimensions. Race and gender were selected as they are easily picturable and have been demonstrated to be salient social properties that influence children’s social inferences early in development (e.g., deMayo and Olson, 2024). Participants were asked a series of forced choice quantitative questions about the group’s potential and their success in the competition. Forced choice measures are ideal as they yield participants’ explicit evaluations of each group. Additionally, participants were required to explain their reasoning to gain insights into the explanatory principles that guided their decisions. Thus, we asked open ended questions about the groups, and had participants explain some of their forced choice answers.

Finally, in our exploratory analyses, we examined how participants’ identities might be influencing their thinking. These exploratory analyses are compatible with our broader aims of identifying the role of experience on people’s thinking about the benefits of diverse groups.

In summary, the goals of the current study are to (1) determine the degree to which 5-to-8-year-olds and adults use diversity to make inferences about their cooperative and innovative potential; (2) assess developmental trends that emerge across age; and (3) determine how participants’ own race and gender identity influences their reasoning about diverse groups. Based on current societal messaging, we predicted that adults would infer that the diverse group has more innovative potential, while the homogenous group would have more cooperative potential. Our predictions for children were less directional. Children may show a similar pattern to adults or may have a general preference for the homogenous ingroup. Either result would suggest an interesting developmental trends. If the former, this would suggest that current societal messages reflect early emerging beliefs about the value of diversity. If the latter, it would imply that children prefer homogeneous ingroups because they develop a strong early sense of group identity that emphasizes similarities among members. Consequently, societal efforts to promote the benefits diversity are having an impact on peoples’ reasoning about diversity and should be implemented at even younger ages. Finally, whether participants own race and gender identity played a role in their reasoning was an exploratory question. We predicted that participants from equity seeking backgrounds may show a different pattern of beliefs, but did not have clear directional hypotheses.

## Materials and methods

### Experiment 1 (adults)

#### Participants

Hundred participants (48 Men, 49 Woman, 3 unknown) completed Experiment 1 through the online crowdsourcing platform Prolific and were from White (64) and East Asian (33) backgrounds. Participants were living in Canada (56) and the United States (41). Using the screening options available only participants from White or East Asian backgrounds (mutually exclusive categories) were eligible to sign up for the study. We included both White and East Asian participants as both groups were reflected in the experimental stimuli, and we wanted to ensure that we collected the perspectives of not only White adults. Additionally, using the screening option, only participants fluent in English were eligible. All participants were fluent in English and the majority identified as monolingual English speakers (64). There were no age criteria. Our only exclusion criteria was a comprehension check during the vignette presentation (participants needed to indicate that groups were competing to design a toy), but all participants correctly answered this question. Demographic information for three participants was unavailable, their data is included in the main analyses but excluded from exploratory analyses. The study was approved by Dalhousie University’s ethical review board (REB#2021–5,835). Informed consent was necessary to begin the experiment, and participants were debriefed on the nature of the study following completion and received £1.50.

#### Stimuli

##### Vignette

The first portion of the task comprised of a vignette about a toy competition, with accompanying images to feel more like a story. Note, the vignette was designed such that 5-to-8-year-olds children would be able to comprehend. Visuals accompanied each portion of the vignette and the script appeared at the top of screen (see https://osf.io/5v9hs/?view_only=858f810e5b1d4910b69f49a11b31f893 for all stimuli and scripts).

##### Visual stimuli

**Figure 1:**
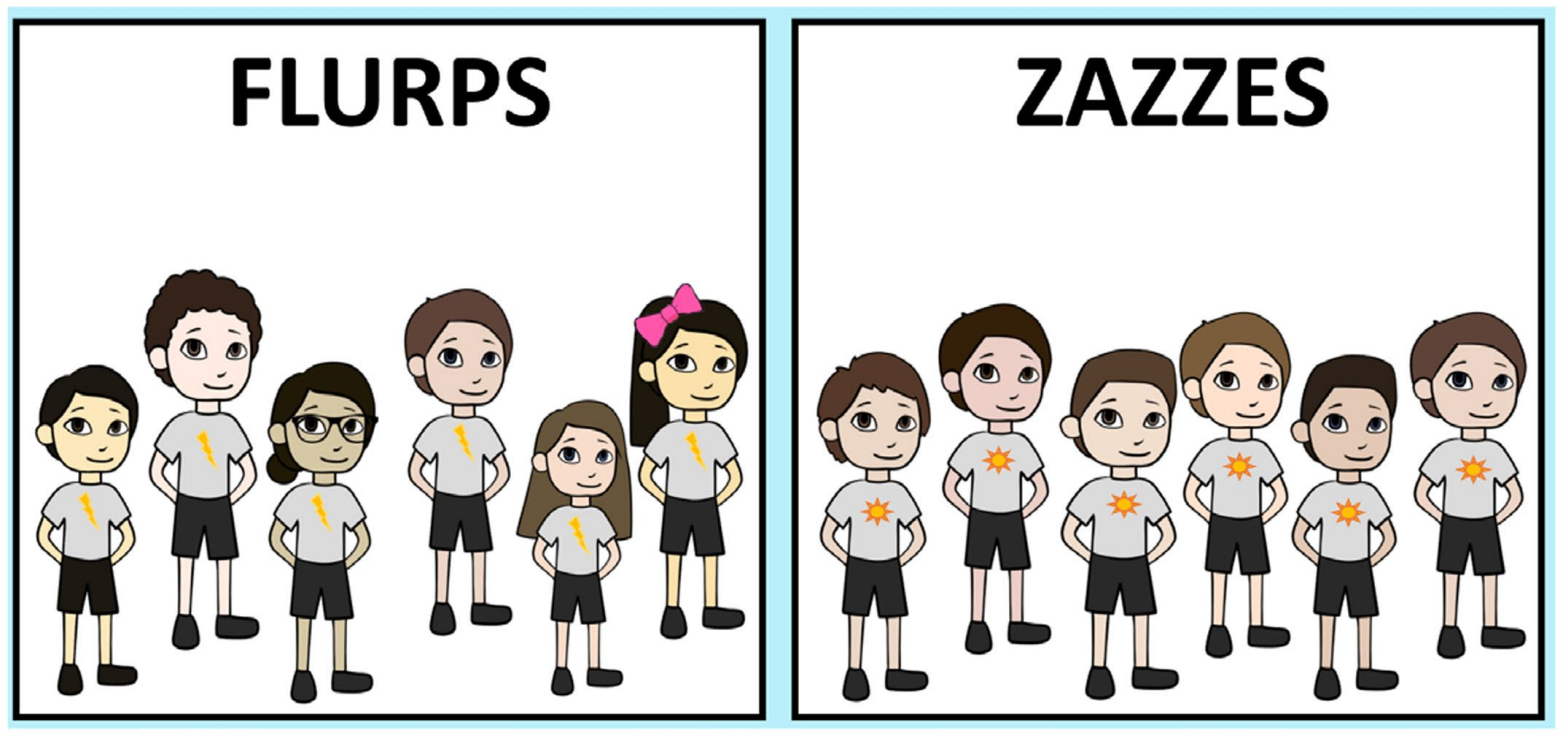
Flurps and Zazzes stimuli for adult participants. Depiction of stimuli presented to a White man. Diverse groups (left) and homogenous groups (right).

Visual stimuli consisted of groups of custom designed cartoon characters that varied on race and gender. For the first question block (questions 1 and 2), the *homogenous group* was comprised of six same-race and same-gendered individuals as the participant (e.g., if the participant was a White woman, she saw six White girls). The *diverse group* was comprised of six characters that differed in race and gender (two White boys, one East Asian boy, one White girl, and two East Asian girls). These characters also varied more on other properties such as height and accessories. Both groups were presented wearing a neutral-colored shirt in a neutral stance. Each group had a symbol on their shirt to reinforce that they were members of the same team (see Figure 1). For the second question block (questions 3 and 4), participants saw two different teams, one homogenous and the other diverse, signified through a change in group size and shirt symbols. The homogenous group was again comprised of same-race and same-gendered individuals as the participant. The diverse group was comprised of characters that differ in gender and race (one White girl, one East Asian girl, one White boy, one East Asian boy).

#### Procedure

The study was created using Qualtrics and disseminated using Prolific. Participants were first presented with a story about the toy competition in which various groups of different sizes and compositions (i.e., varying degrees of diversity) were competing to design the perfect toy.

A comprehension check was added to ensure the participants were attentive, asking them, “What are the groups competing to make?” Participants typed their responses in a text box. Once participants had responded to the comprehension check, they were introduced to various groups eligible to enter the contest. The stimuli presented to the participants featured four different groups varying in diversity levels and group size. Participants were informed that each group had one table to work at, one box of supplies, and that all groups were provided with the same supplies.

In the next part of the study, participants were introduced to the diverse and homogenous group of the first question block one at a time (e.g., “Here is one group, the Flurps”; Note both groups were size matched and contained six members). They were then told the group was working hard together to think of the best idea for the toy and that they are having lots and lots of good ideas. Participants were then asked to explain why the group had good ideas (e.g., “Why do you think the Flurps have so many good ideas?”). Participants wrote their explanations in a text box. This was done for both the homogenous and diverse group.

After participants were familiar with both groups, they were presented with the diverse and the homogeneous group side by side. Participants were prompted, “Which team made the best toy?.” Participants made their selection by clicking on the picture of the group. They were then prompted to explain why they chose that group. The same process was repeated with a different question, “Which team made their toy the fastest?” (order counterbalanced).

Participants were then introduced to a character named Eric, or Erica for female participants. The race and gender of this character was matched with the participant. They also saw two novel groups above the character on either side of the screen, one homogenous [gender/race matched to Eric(a)] and one diverse. These groups were not the same as the Flurps/Zazzes previously presented, the groups were not highlighted to the participant in any way. Participants were asked which team Eric(a) should join so they can win the contest. Participants could choose from the novel homogeneous and diverse group presented to them. Finally, participants were instructed to pretend that they were in the contest and asked which group they thought would be the “hardest to beat.”

Group names and the order in which the groups were presented were counterbalanced. Participants were monetarily compensated through prolific.

#### Coding

##### Forced choice data

Quantitative data was collected for four forced-choice questions:

1. Which group made the best toy? (Best Toy)
2. Which group made their toy the fastest? (Fastest Toy)
3. Which group should Eric(a) join? (Join Team)
4. Which group will be hardest to beat? (Hardest to Beat)

Data was sum-coded, such that selecting the diverse group were coded as 1 and selecting the homogenous group was coded as 0.

The Best Toy question probes participants beliefs about innovative potential, as it focuses on the overall quality of the group’s final product. The Fastest Toy question probes beliefs about cooperative potential, as group cooperation and harmony are necessary to produce an output quickly. The final two questions address beliefs about the overall strength of the group. The Join Team question addresses participants preferences between the two groups, while the Hardest to Beat question addresses beliefs about the overall quality of the team. Thus, while the final two questions probe beliefs about the quality of the group, they are not related to the innovative or cooperative potential of the group.

##### Explanation data

Explanation data was collected for four questions:

1. Why do you think the [diverse group] has so many good ideas?
2. Why do you think the [homogenous group] has so many good ideas?
3. Why do you think the [selected team] made the best toy?
4. Why do you think the [selected team] made their toy the fastest?

Two coders blind to the research questions of this study as well as participants forced choice selection evaluated each explanation and categorized them into two main categories: Group Dynamics and Mental Processes (see Table 1). Theoretical models of innovation suggest that cognitive resources, that is the amount of knowledge, skills, values, and beliefs, increases a groups innovative potential (Wanous and Youtz, 1986; Bantel and Jackson, 1989). A measure of perceived cognitive resources is captured by the Mental Processes category. At the same time, the innovative potential of diverse teams is also dependent on people’s willingness to cooperate (Roh and Koo, 2019). A measure of perceived cooperation is captured by the Group Dynamics category. Note these were not mutually exclusive categories, thus explanations can fall into more than one main category (see OSF for full coding manual).

**Table 1:** Description of the coding categories, including examples.

Explanation category	Description	Examples	Kappa (Adults)	Kappa (Children)
Group dynamics	Explanations in this category focus on the group’s ability to work with each other.	“… stronger individuals, possibly the men were able to make the toy more efficiently. While they put together heavier stuff, others might’ve been able to multi-task.” “…it seems like they are all females who tend to be more empathetic and active listeners. Being more willing to hear each other’s ideas in a less competitive way will generative a collaborate idea generating culture.”	*κ* = 0.76 (0.63–0.89)	*κ* = 0.88 (0.80–0.96)
Mental processes	Explanations in this category focus on the group’s cognitive resources. Explanation might reference people having better ideas, being more intelligent, or thinking hard.	“There are many of them and they are all creative, I assume as they formed a group to make the best toy of the smartest most creative people they could find.” “I think that they are smart and creative. I think that they understand that this toy needs to appeal to many different groups of people, and are coming up with ways to satisfy that…”	*κ* = 1.00 (1.00–1.00)	*κ* = 0.91 (0.87–97)

Cohen’s Weighted Kappa was calculated for both experiments using the statistics program JASP (Team JASP, 2019). These scores indicated substantial to almost perfect agreement between two independent blind coders. Any disagreements were resolved through discussion. These categories were not mutually exclusive. If participants’ explanations did not fall into one of these two categories, coders could code the explanation as Visual Information (which only touch on visual information in the scene), I Do not Know, No Response, or Other. These other categories were not analyzed statistically.

##### Group dynamics

Explanations in this category focus on the group’s ability to work with each other. Examples include:

“Depending on the size of the toy, maybe the stronger individuals, possibly the men were able to make the toy more efficiently. While they put together heavier stuff, others might’ve been able to multi-task.”

“Based on the team’s appearance, it seems like they are all females who tend to be more empathetic and active listeners. Being more willing to hear each other’s ideas in a less competitive way will generative a collaborate idea generating culture.”

##### Mental processes

Explanations in this category focus on the group’s mental processes. For example, it might reference people having better ideas, being more intelligent, or thinking hard. Examples of Mental Processes:

“There are many of them (6). and they are all creative, I assume as they formed a group to make the best toy of the smartest most creative people they could find.”

“I think that they are smart and creative. I think that they understand that this toy needs to appeal to many different groups of people, and are coming up with ways to satisfy that, despite their more similar background.”

Examples of overlapping explanations:

“They have open communication and collaboration. The group does not have egos, so everyone’s idea is treated equally. There is a lot of diversity in the group which allows for many different sources of inspiration for ideas.”

“They are creative. They help each other to think creatively. They are collaborative. Some of them have smartphones and are using Google. They use keywords like “toys made out of… “And then list the random items that are on the table. They also type into Google “most popular toys in history” for more ideas. They also use the search engine Yandex in case Google is censoring them. More search engines equal more ideas.”

There were also “Other” Categories: Visual information (referred to non-social information in the stimuli, e.g., “Their shirt is a bolt of lightning”), “I do not know,” No response, and other (unintelligible or uninterpretable). For full information please see the supplementary materials in Open Science Framework.

### Results

#### Planned analyses

#### Forced choice data

Of key interest to the current study is the difference between participants response to the Best Toy and Fastest Toy, because these questions were specifically designed to probe participants beliefs about innovative and cooperative potential. A binomial regression with within factor Question (Best Toy, Fastest Toy) was run using R (Posit Team, 2023). As predicted, a significant main effect of Question was found, *b_2_ =* −2.09*, confidence interval (CI) =* −2.74 to −1.45*, z =* −6.3*, p <* 0.001, *OR =* 0.12, indicating that participants were endorsing the diverse group more when asked about the Best Toy as compared to Fastest Toy (see https://osf.io/5v9hs/?view_only=858f810e5b1d4910b69f49a11b31f893 for all data).

**Figure 2:**
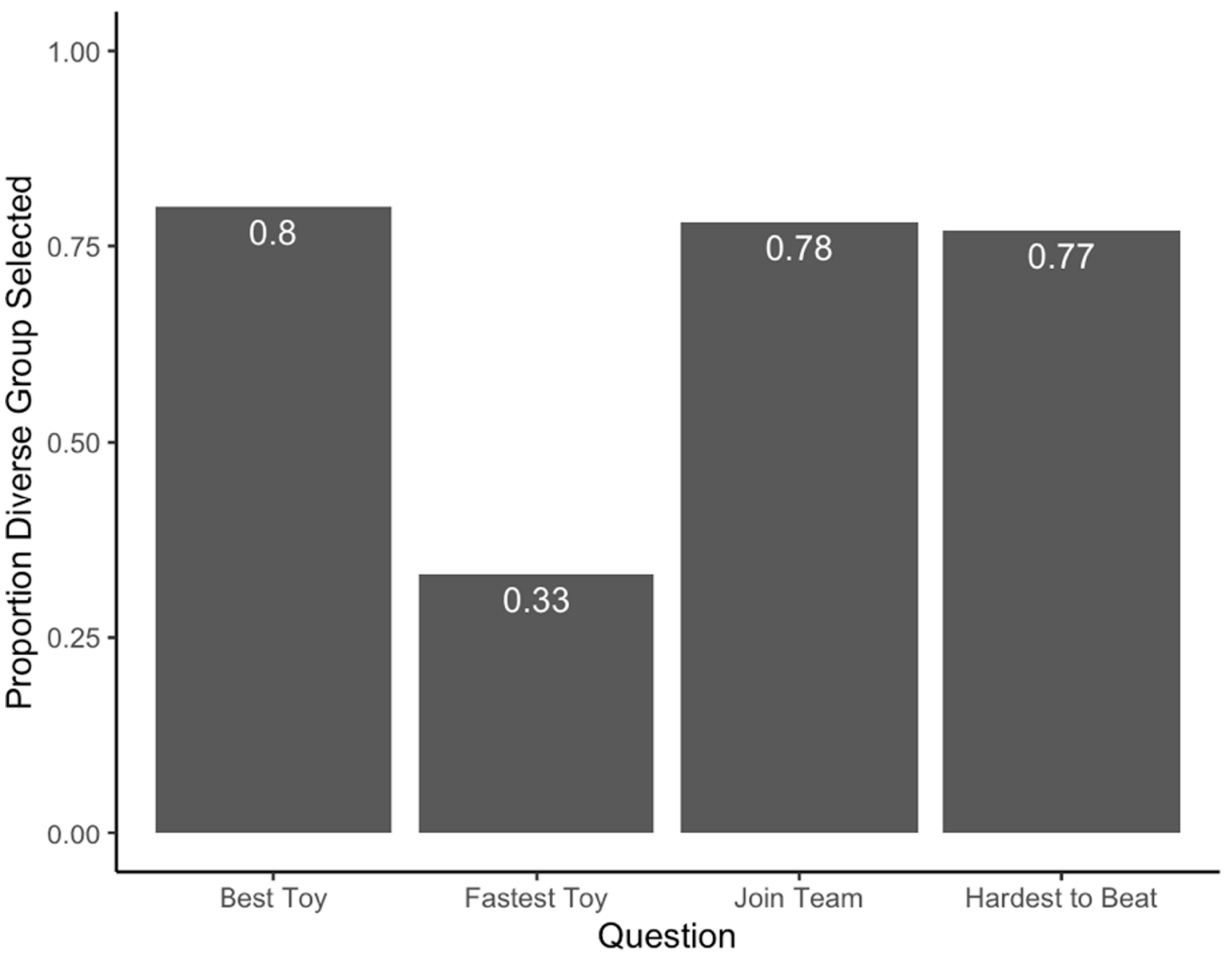
Adults’ forced choice responses. Mean scores for each quantitative question. *X* axis indicates the question asked, *Y* axis indicates the proportion of responses that selected the diverse Group.

Next, a binomial test against chance was run for each of the four quantitative questions (see Figure 2) to determine whether participants were more likely to endorse the diverse or homogenous group in comparison to chance. For the *best toy*, participants selected the diverse group above chance rates, *CI* = 0.71–0.87, *p* < 0.001. For making the toy the *fastest*, participants selected the homogenous group above chance rates, *CI* = 0.24–0.43, *p* < 0.001. These two results suggest that participants endorsed the diverse group more in the question probing innovative potential, and the homogenous group more in the question probing cooperative potential. For which team Eric(a) *should join*, participants selected the diverse group at above chance rates, *CI* = 0.68–0.85, *p* < 0.001. Finally, for the *hardest team to beat*, participants selected the diverse group at above chance rates, *CI* = 0.66–0.84, *p* < 0.001. These two results suggest an overall preference for the diverse group when reasoning about the strength of the group overall.

##### Explanation data

For the explanation data, we were interested in whether the principle(s) or the factor guiding participants reasoning differed depending on their endorsement of the diverse or homogenous groups for each question (see Table 2).

**Table 2:** Descriptive summary for each explanation category.

Question	Explanation type	Experiment 1 (Adults)		Experiment 2 (Children)	
		Diverse group	Homogenous group	Diverse group	Homogenous group
Best toy (Why)	Group dynamics	0.18	0.40	0.14	0.09
	Mental processes	0.40	0.30	0.31	0.38
Fastest toy (Why)	Group dynamics	0.30	0.66	0.12	0.04
	Mental processes	0.27	0.31	0.22	0.22
Good ideas? (Why)	Group dynamics	0.33	0.45	0.11	0.11
	Mental processes	0.78	0.72	0.39	0.39

Explanation categories presented as a function of whether the explanation was about a diverse or homogenous group (proportions).

Participants were split based on whether they selected the homogenous or diverse group for each question. The rate at which they mentioned Group Dynamics, Mental Processes, or Both in their explanations was compared (to ensure mutually exclusive categories) for each question using individual *X^2^* tests of independence or Fisher Exact Tests (dependent on sample size). For Best Toy, participants who endorsed the diverse group were more likely to refer to Mental Processes than Group Dynamics or Both, to explain their thinking, *X^2^* (2, 80) = 21.48, *p* < 0.001, but those that endorsed the homogenous group did not show these differences, *p* = 0.265. For the Fastest Toy, participants who endorsed the homogenous group were more likely to refer to Group Dynamics than Mental processes or Both, *X^2^* (2, 67) = 21.12, *p* < 0.001, but those that endorsed the diverse group did not show these differences, *p* = 0.256. Thus, adults’ explanations differed depending on whether they endorsed the diverse or homogenous group. In the innovation question adults who selected the diverse group were more likely to be considering mental processes, and in the cooperation question adults who selected the homogenous group were more likely to refer to group dynamics.

#### Exploratory analyses

**Figure 3:**
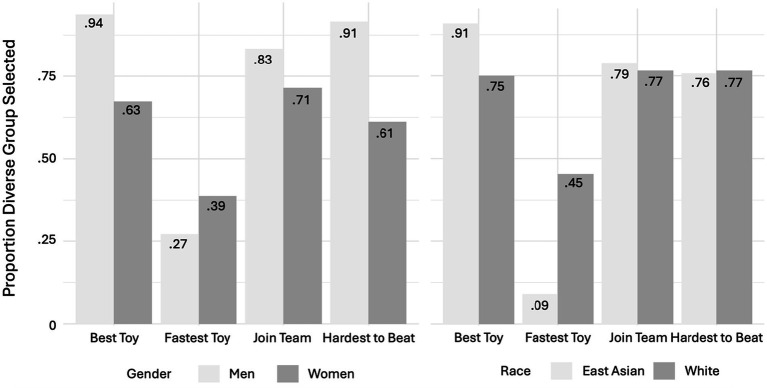
Adults’ responses to the Forced Choice Questions as a function of Participant Gender and Race. Mean scores for each quantitative question as a function of Gender (Men or Women; Left) and Race (East Asian or White; Right). *X* axis indicates the question asked, *Y* axis indicates the proportion of responses that selected the diverse Group.

To explore the potential roles of participants’ gender and race, a binomial regression was run using R (Posit Team, 2023) with the within factor Question (Best Toy, Fastest Toy) and between subject factors participant Gender (Men, Women) and participant Race (East Asian, White), as well as the Gender*Question and Race*Question interactions. A significant main effect of Question was observed, *b_2_ =* 2.99*, CI =* 1.96–4.02*, z =* −5.71*, p <* 0.001, *OR =* 19.89. A significant Race*Question interaction was found, *b_2_ =* −1.59*, CI =* −2.53- -0.65*, z =* −3.32*, p <* 0.001, *OR = 0*.20, as well as a significant Gender*Question interaction, *b_2_ =* −1.17*, CI =* −1.98 to 0.36*, z =* −2.84*, p =* 0.004, *OR = 0*.31. These results indicate that how participants responded to the Best Toy and Fastest Toy questions differed as a function of both participant Gender and participant Race (see Figure 3).

##### By gender

First, we followed-up on participant Gender effects using a binomial regression with the within factor Question (Best Toy, Fastest Toy) and between subject factors Gender (Men, Women) to determine if participant gender played a role in adults’ reasoning about the innovative and cooperative potential of the groups. A significant main effect of Question was observed, *b_2_ =* 1.86*, CI =* 1.93 to 1.52*, z =* 2.53*, p =* 0.011, *OR =* 6.35. A significant Gender*Question interaction was found, *b_2_ =* −1.26*, CI =* −2.04 to −0.48*, z = −*1.26*, p =* 0.002, *OR = 0*.28, suggesting that gender impacted participants beliefs about the groups’ potentials.

To determine the direction of this effect, two-sample tests were run comparing men and women’s responses by question using individual *X^2^* tests of independence/Fisher Exact Tests. For the Best Toy question, men were more likely to choose the diverse group than women, *p* = 0.002. However, for the Fastest Toy question, there were no gender differences*, p* = 0.313. Based on Figure 3, the Hardest to Beat question was further analyzed, and found men were more likely to choose the diverse group than women, *p* = 0.001. In sum, women may have been more likely to value in-group homogeneity in questions probing innovative potential.

##### By race

A binomial regression was run with the within factor Question (Best Toy, Fastest Toy) and between subject factors participant Race (East Asian, White) to determine if participants’ race played a role in adults’ reasoning about the innovative and cooperative potential of the groups. A significant main effect of Question was observed, *b_2_ = 0*.02*, CI =* 1.46 to 3.14*, z =* 5.38*, p <* 0.001, *OR =* 2.35. A significant Race*Question interaction was found, *b_2_ =* −1.76e^00^*, CI =* −2.58 to −0.74*, z = −*3.54*, p =* 0.004, *OR = 0*.28, suggesting that race impacted participants beliefs about the groups’ potentials.

To further examine these effects, Fisher Exact tests were run comparing East Asian and White participants responses in the Best Toy and Fastest Toy forced choice questions. For the Fastest Toy question, East Asian participants were more likely to choose the homogenous group than White participants, *p* < 0.001. However, for the Best Toy question, there was no difference found*, p* = 0.103. In sum, East Asian participants might be more likely to value homogeneity when probed about cooperative potential.

### Experiment 2 (children)

Experiment 2 aimed to map development in people’s thinking about diverse groups by examining children’s decisions and explanations. There were at least three possible outcomes with children. First, they might show similar reasoning to adults, and thus endorse that diverse groups have better ideas but, homogenous groups work faster. Second, children might prefer homogenous groups due to strong prescriptive beliefs that group members should be similar, and strong ingroup preferences as the homogenous group included more ingroup members (Roberts et al., 2017a, 2017b). Indeed, both of those factors are ecologically valid as they would compete with children’s intuitions in “real-world” settings. Third, there might be age-related changes where young children might perform as predicted in (2) and older children as predicted in (1).

#### Participants

Hundred 5-to-8-year-olds participated in this virtual study from across Canada: (*M*_age_ = 7.12, Range: 5.03:8.94, 44 girls, 56 boys). An additional 11 children participated but their data was not included in the final sample due to parental/sibling interference (7), failing to answer 50% or more of the questions (3), and technical issues (1). Families were recruited through online and in-person advertisements from across Canada, and reported being from White (46), South Asian (19), East Asian (14), Black/African Canadian or American (2), and Multiracial or Other (19) backgrounds. There were no language criteria for this study except that children must be fluent in English. Sixty-five participants were reported as having at least some exposure to another language in addition to English.

The study was approved by Dalhousie University’s ethical review board (REB#2022–6,144) and carried out in accordance with the Declaration of Helsinki and APA ethical standards; primary caretakers completed an informed consent form prior to families taking part in the study. Participants completed this study first in a series of three unrelated experiments and were gifted a $10 Amazon gift card.

The method was very similar to Experiment 1 with a few exceptions to make the design even more developmentally appropriate. The differences are outlined below.

#### Stimuli

##### Warm up task

Eight clip art images were created in Microsoft PowerPoint. For both trials, 4 images appeared in each corner of the screen (Trial one: broccoli, ice cream, pizza, carrots; Trial two: bird, cat, dog, rabbit).

##### Main task

**Figure 4:**
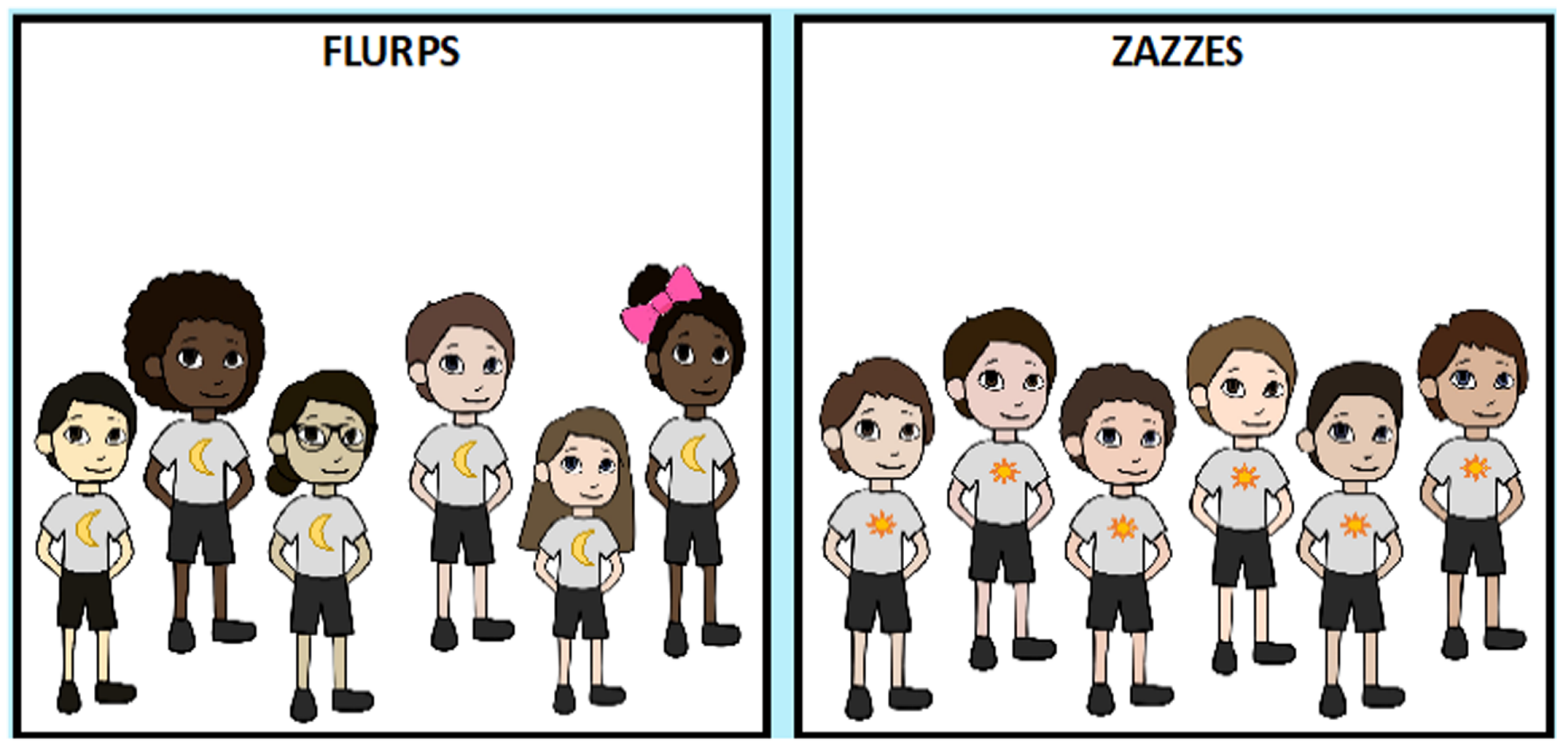
Flurps and Zazzes stimuli for child participants. Depiction of stimuli presented to a White boy. Diverse groups (left) and homogenous groups (right).

In comparison to Experiment 1, the diverse group consisted of more racial diversity, including Black, East Asian, and White characters (see Figure 4). This was done because our sample was expected to be more racially diverse. As in Experiment 1, the *homogenous group* for the first question block was comprised of six same-race and same-gendered individuals as the participant. If a participant did not identify as White, Black, East Asian, or South Asian, they saw a gender-matched group of White children as White is the majority race in all demographic regions tested (when parents were asked explicitly which race was most familiar to the child, almost all said White). The *diverse group* for the second question block was comprised of six characters that differed in race and gender (e.g., one White boy, one East Asian boy, one Black boy, one White girl, one East Asian girl, and one Black girl). For the second question block, the homogenous group was again gender and race-matched to the participant, while the diverse group remained consistent from Experiment 1.

#### Procedure

This study was conducted remotely through Zoom and Microsoft Teams using screen-sharing with a Microsoft PowerPoint to present stimuli to the participants. Parents could accompany their children but were instructed not to interfere in answering questions. A simple warm-up game was presented to the participants, consisting of visual stimuli organized into two distinct categories (food and animals). Participants were asked to state which items they liked, did not like, and why.

Following the warm-up activity, participants were introduced to the experiment. The experimenter read the vignette and asked all experimental questions orally. Participants verbally gave their responses. If the participant had not spoken within 10 s of the questions being asked, the experimenter would repeat the question. If the participant seemed hesitant or did not respond at this point, the experimenter would encourage the participant to take their best guess. If the participant had not responded after three attempts, the experimenter would skip that question and continue the study. For the child sample, the position of the group on the screen (left or right) and groups shirt symbols were additionally counterbalanced.

### Results

#### Coding

Refer to Table 1 for Kappa’s and coding scheme.

Examples of Group Dynamics explanations:

“Because they work together.”

“Because they worked together and knew what to build.”

Examples of Mental Processes explanations:

“Because they are thinking.”

“Because they are using their imaginations.”

“Because they are good thinkers.”

“Because, mhmmm, they are just thinking of good ideas.”

“Because they have smart brains.”

#### Planned analyses

##### Forced choice data

Again, of key interest to the current study is the difference between participants response to the Best Toy and Fastest Toy, because these questions were specifically designed to probe participants beliefs about innovative and cooperative potential. A binomial regression with within factor Question (Best Toy, Fastest Toy; contrast coded) and Age (as centered, continuous variable) was run using R (Posit Team, 2023). A significant main effect of Question *b^2^* = −0.29, *(CI) =* −0.57 to –0.01, *Z* = −2.00, *p* = 0.046. *OR =* 0.75, with participants endorsing the homogenous group more when asked about the Best Toy as compared to Fastest Toy. No main effect of Age, *p* = 0.476, nor a Question*Age interaction, *p* = 0.707, was observed.

**Figure 5:**
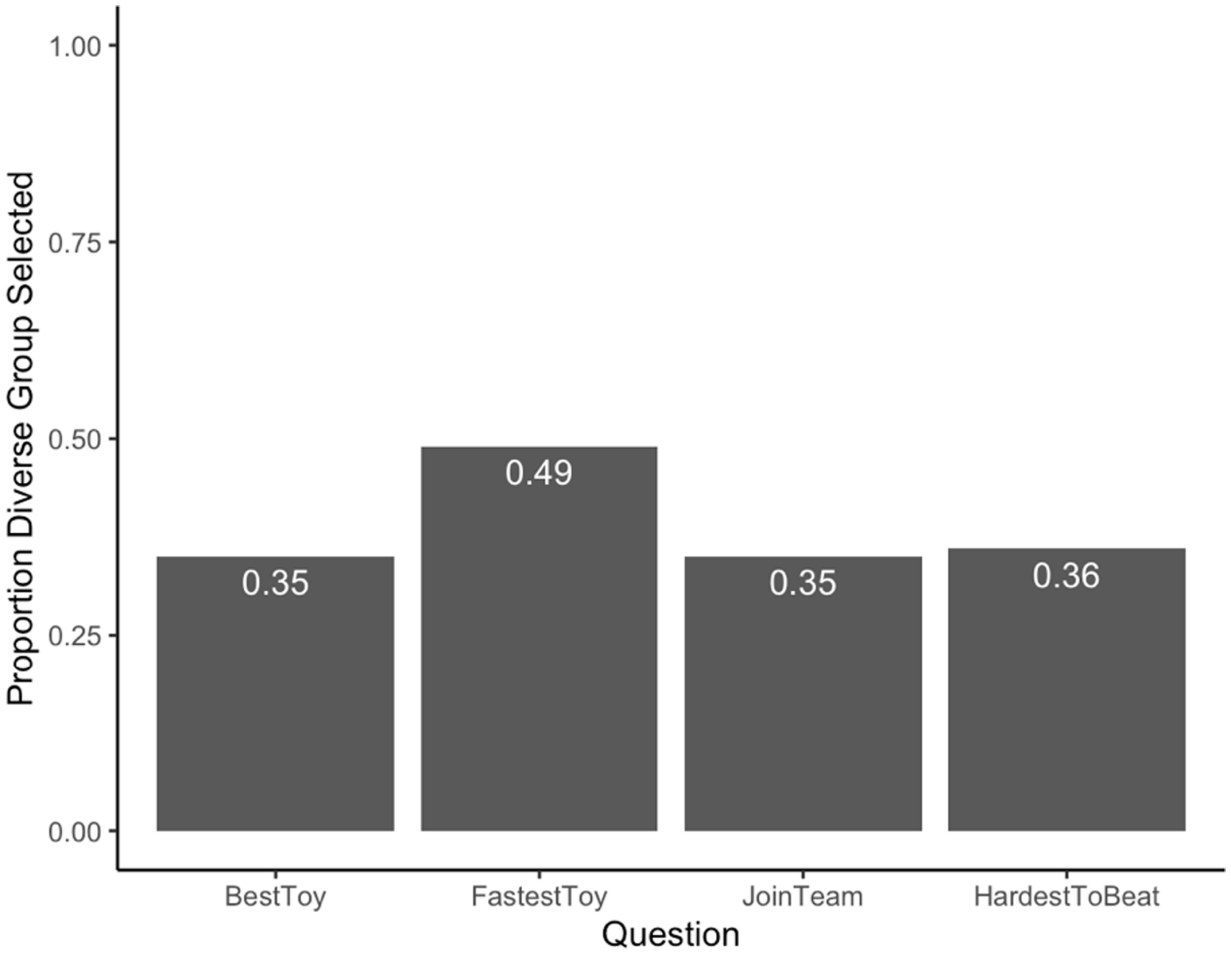
Children’s forced choice responses. Mean scores for each quantitative question. *X* axis indicates the question asked, *Y* axis indicates the proportion of responses that selected the diverse Group.

Next, binomial tests against chance were run for each of the four quantitative questions to determine whether participants were more likely to endorse the diverse or homogenous group in comparison to chance (see Figure 5). When asked which group made the *best toy*, participants selected the homogenous groups at rates above chance *CI* = 0.26–0.45, *p* = 0.004. When asked which group made their toy the fastest, participants’ responses did not favor either group *CI* = 0.39–0.60, *p* = 1.00. These two results suggest that children endorsed the homogenous group more when probed about innovative potential, and neither group more than the other when probed about cooperative potential. When asked which team Eric(a) should join, participants selected the homogenous group at rates above chance, *CI* = 0.26–0.45, *p* = 0.004. Finally, when asked which team would be the hardest team to beat, participants selected homogenous group at rates above chance, *CI* = 0.27–0.46, *p* = 0.007. These two results suggest an overall preference for the homogenous group when reasoning about the strength of the group overall.

##### Explanation data

In general, child participants struggled with providing explanations (see Table 2), with most of their responses falling into the “Other” categories. Participants were split based on whether they selected the homogenous or diverse group for each question then the rate at which they mentioned Group Dynamics, Mental Processes or Both was compared using Fisher Exact Tests (Note: the only cases in which both were referenced was when justifying why they selected the diverse group). For Best Toy, participants who endorsed the homogenous group were more likely to refer to Mental Processes than Group Dynamics, to explain their thinking, *p* < 0.001, but those that endorsed the diverse group did not show any difference across explanation type, *p* = 0.115. Thus, for the Best Toy question children were more likely to endorse the homogenous group (based on the forced choice data) and were more likely to reference Mental Processes when they selected the homogenous group. For the Fastest Toy, children were more likely to reference Mental Processes regardless of whether they endorsed the homogenous or diverse Group, *p*s < 0.017. Thus, for the Fastest Toy question children did not endorse one group more than the other (based on the forced choice data) and were more likely to reference Mental Processes regardless of the group they chose. Overall, across both question types there was relative low rates of discussing Group Dynamics. These results suggest there is some nuance in children’s explanations, though not to the same degree as adults. In the innovation question, children who selected the homogenous group were more likely to consider mental processes. However, in the cooperation question, they considered mental processes more, regardless of which group they selected.

#### Exploratory analyses

To explore the potential roles of participant gender and race, a binomial regression was run using R with the within factor Question (Best Toy, Fastest Toy; contrast coded) and factors participant Gender (Boy, Girl) and participant Race (Minority Race, White), as well as Gender*Question and Race*Question interactions. Due to the varying racial backgrounds of the children, we compared Majority race (White) children a group of Minority race children that included children from an East Asian, Black, South Asian, and Multiracial or Other background. No significant effects were found, *p*s > 0.356.

**Figure 6:**
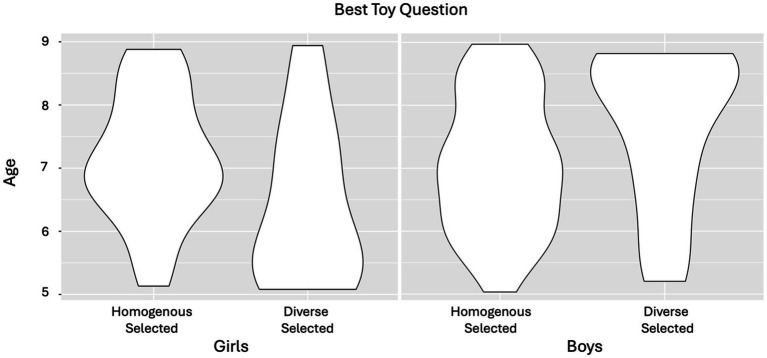
Children’s responses to the Best Toy Question as a function of Participant Gender and Age. Girls’ and Boys’ selection of the homogenous and diverse group in an innovation question as a function of age (*y*-axis).

To follow up on the results observed in the exploratory analyses for Experiment 1, a binomial regression for the scores in the Best Toy question was run as a function of participant Gender (Boy, Girl) and Age (centered, as a continuous factor), and a binomial regression for the scores in the Fastest Toy question as a function of participant Race (Minority, White) and Age (centered, as a continuous factor). In the first model, a significant Gender*Age interaction was found, *b^2^* = −1.07, *(CI) =* −1.87 to –0.26, *Z* = −2.60, *p* = 0.009, *OR* = 0.34; however, no significant effects were observed in the second model, suggesting that while children’s gender may play a role in their reasoning, participant race did not. Thus, separate models were run for girls and boys with Age as a predictor (see Figure 6). For girl participants a main effect of age was observed, *b^2^* = −0.61, *(CI) =* −1.24–0.02*, Z* = 1.90, *p* = 0.058, *OR* = 0.54. For boys there was a marginal but non-significant effect of age, *b^2^* = 0.45, *(CI) =* −0.04 to 0.95*, Z* = 1.79, *p* = 0.074, *OR* = 1.58. These results suggest girls became more likely to select the homogenous group when probed about innovative potential with age, consistent with the result observed in women in Experiment 1, and boys become more likely to select the diverse group when probed about innovative potential with age, consistent with the men in Experiment 1.

## Discussion

In two experiments we map adults’ and children’s thinking about the potential of diverse and homogenous groups for innovation and cooperation. When probed about innovation potential (Best Toy), adults (Experiment 1) believed that the diverse group would produce the best toy, while children (Experiment 2) endorsed the homogenous group. When probed about cooperative potential (Fastest Toy), adults overwhelmingly selected the homogenous group when asked which group made their toy the fastest. In contrast children were equally likely to select either group. These results suggest that people’s appreciation of the link between group diversity and potential changes across the lifespan likely due to life experiences. In support of an experience-based account of people’s appreciation of diverse groups, individual differences in people’s endorsements were detected in the exploratory analyses.

An analysis of adults’ explanations showed that their explanations differed depending on whether they endorsed the diverse or homogenous group. For example, in the innovation question adults who selected the diverse group were more likely to be considering mental processes, and in the cooperation question adults who selected the homogenous group were more likely to refer to group dynamics. Children too showed some nuance in their explanations, though not to the same degree. In the innovation question, children who selected the homogenous group were more likely to consider mental processes. However, in the cooperation question, they considered mental processes more, regardless of which group they selected.

Taken together, these results suggest that adults tended to believe that diverse groups have greater innovative potential than homogenous groups, because their collective knowledge will lead to better ideas. This result is reinforced by the two additional forced choice questions in which adults reasoned that if someone wanted to win the competition, they would be more successful joining a diverse group, and that if they personally wanted to win the competition it would be harder to beat a diverse group. In contrast, adults reason that homogenous groups have more cooperative potential than diverse groups. Adults’ intuitive theories of the benefits of diverse and homogenous groups align with real world findings. Namely, work demonstrating that more demographically diverse groups tend to engage in more innovative behavior (Bantel and Jackson, 1989). Adults’ beliefs also align with cognitive theories of innovative potential suggesting that diverse groups are more innovative because of their increased cognitive resources (Bantel and Jackson, 1989; Jackson and Ruderman, 1995) and creative potential (Ruiz-Jiménez et al., 2016). This suggests that real world messaging about the importance of diversity is having an impact on how adults view and discuss diversity in team settings.

In comparison, children believe that homogenous groups have more innovative potential than diverse groups. This result is reinforced by the two additional forced choice questions in which children reasoned that if someone wanted to win the competition, they would be more successful joining a homogenous group, and that if they personally wanted to win the competition it would be harder to beat a homogenous group. This result is not entirely unsurprising given the extensive research suggesting that children prefer ingroup members over outgroup members (Aboud, 1988; Kinzler et al., 2007; Dunham, 2018; deMayo and Olson, 2024). Furthermore, children’s essentialist beliefs toward out-group members are also strengthening during the developmental period tested here (Levy and Dweck, 1999; Pauker et al., 2010; Gaither et al., 2014). In general, children seemed to have a bias to prioritize mental processes over group dynamics in their explanations. However, in the innovation question they only prioritized mental processes more than group dynamics if they selected the homogenous group. This behavior suggests that children may have intuitive theories about group innovation. Namely, that a group’s innovative potential hinges on their cognitive resources (Bantel and Jackson, 1989; Jackson and Ruderman, 1995). However, the current results suggest that children are not yet considering the impact of demographic diversity on a group’s collective cognitive resources.

The exploratory analyses enrich our interpretation of the data as well. For both children and adults, participants’ gender influenced responses in the forced choice questions. Women appeared to value in-group homogeneity in an innovative question (Best Toy and Hardest to Beat). Based on the child data, this is a trend that seems to emerge throughout childhood. For adults only, race also appeared to play a role in participants’ behavior. Specifically, East Asian participants appeared to value homogeneity when probed about cooperative potential. Perhaps it is unsurprising that adults from an underrepresented race and/or gender may be more likely to value in-groups in particular contexts, as they are more likely to have had unpleasant experiences as a member of diverse groups (Triana et al., 2015; Sojo et al., 2016). For example, women’s experiences with sexism, and perceived workplace tolerance of sexism, can influence how close their relationships are with other women in the workplace (Ciftci et al., 2020). Girls’ increasing preference for ingroup homogeneity may be an important contributing factor when trying to understand gender divisions in scientific fields (e.g., Leslie et al., 2015; Mulvey and Irvin, 2018; Weisgram et al., 2010). Their preference to innovate in homogenous groups may limit the field in which they consider working/studying in.

Overall, these exploratory observations suggest that participants’ responses in the task may be influenced by their personal experiences with gender and racial diversity. A more carefully controlled study with a larger sample size would be required to fully determine the extent to which these factors influence reasoning about innovative and cooperative potential. A larger sample would also allow for the examination of participant diversity in a more intersectional manner (Lei and Rhodes, 2021). Additionally, other factors such as the participants experience with diversity and the amount of diversity where they live should also be taken under consideration.

Another limitation of the current study is that children’s explanations were often uninterpretable. In general, children struggled to produce explanations related to why groups function well. In the innovation question, children were more likely to reference mental processes than group dynamics if they selected the homogenous group. However, with the low overall rates of explanations, replication is needed. In future work, researchers should consider providing children with explanations and having them select the best reason, rather than making them generate their own explanations. Given the novelty of the current findings, this paper can be viewed as the foundation for many areas of future research regarding people’s beliefs about diverse groups. Future work may want to look at other areas of potential (e.g., ability to make decisions), the effect of question (e.g., competition vs. cooperation), aspects of group dynamics (e.g., prosociality), isolate the type of diversity (e.g., isolating gender), and whether other aspects of group diversity that are less visually salient have a similar impact (e.g., intelligence or ability). Finally, in the present work, we did not study how participants beliefs about cooperation or innovation relate to their real-world behaviors. Future work may want to address whether (and how) people’s beliefs about diversity influences who they choose to innovate and cooperate with. We found that visually salient characteristics (like race and gender) influenced people’s beliefs about a group’s potential, but whether this also influences people’s behavior is an open question.

In summary, the current study demonstrates clear developmental changes in people’s thinking about the benefits of group diversity and homogeneity across the lifespan. Adults’ explanations are in line with theoretical models of cognitive diversity, whereas children tend to reference cognitive processes. Finally, participants’ life experience as measured by race, gender, and age plays a role in their beliefs about the innovative and cooperative potential of groups.

## Data Availability

The datasets presented in this study can be found in online repositories. Repository link: https://osf.io/5v9hs/?view_only=858f810e5b1d4910b69f49a11b31f893.
